# Seasonal Effects on Bioactive Compounds and Antioxidant Capacity of Six Economically Important *Brassica* Vegetables

**DOI:** 10.3390/molecules16086816

**Published:** 2011-08-10

**Authors:** Alfredo Aires, Conceição Fernandes, Rosa Carvalho, Richard N. Bennett, Maria J. Saavedra, Eduardo A.S. Rosa

**Affiliations:** 1 CITAB⁄UTAD-Centre for the Research and Technology for Agro-Environment and Biological Sciences, Universidade de Trás-os-Montes e Alto Douro, Apartado 1013, 5001-801 Vila Real, Portugal; 2 CIMO/IPB-Mountain Research Centre, Escola Superior Agrária, Instituto Politécnico de Bragança, Campus de Sta Apolónia, Apartado 1172, 5301-855 Bragança, Portugal; 3 Departamento de Agronomia, Universidade de Trás-os-Montes e Alto Douro, Apartado 1013, 5001-801 Vila Real, Portugal; 4 CECAV⁄UTAD-Veterinary and Animal Science Research Center, Departamento de Ciências Veterinárias, Universidade de Trás-os-Montes e Alto Douro, Apartado 1013, 5001-801 Vila Real, Portugal

**Keywords:** *Brassicacea*, phytochemicals, antiradical and antioxidant activity, climate

## Abstract

Research on natural and bioactive compounds is increasingly focused on their effects on human health, but there are unexpectedly few studies evaluating the relationship between climate and natural antioxidants. The aim of this study was analyze the biological role of six different *Brassica* vegetables (*Brassica oleracea* L. and *Brassica rapa* L.) as a natural source of antioxidant compounds. The antioxidant activity may be assigned to high levels of L-ascorbic acid, total phenolics and total flavonoids of each sample. The climate seasons affected directly the concentration of bioactive components and the antioxidant activity. Broccoli inflorescences and Portuguese kale showed high antioxidant activity in Spring-Summer whilst turnip leaves did so in Summer-Winter. The *Brassica* vegetables can provide considerable amounts of bioactive compounds and thus may constitute an important natural source of dietary antioxidants.

## 1. Introduction

The *Brassicaceae* plants are amongst the most consumed vegetables in the World. They feature a large biodiversity, in which landraces and primitive cultivars still play a major role on the cultivation systems of many countries, such as Portugal. These plant products have also been associated with beneficial health effects based on the presence of bioactive compounds, with antioxidant capacity, such as vitamins C and E [1], carotenoids, phenolics, flavonoids [2,3]. Studies on the plant product composition and their potential health effects have gained increase relevance and attracted consumer awareness; however these studies are typically related to the effect of individual compounds and only exceptionally to a matrix of compounds. Consumption of antioxidants, either from natural or synthetic origin, reinforce the protection mechanisms against free-radicals and reactive oxygen species (ROS) [4], however, synthetic antioxidants have several restrictions due their potential carcinogenic risk, when used during prolonged periods [5], which has prompted increase interest in antioxidant compounds from natural sources. 

Antioxidant compounds are being extensively researched due to their potent antioxidant activity, their ability to wipe up harmful free radicals, and the associated health benefits. Many have also been implicated in possible protection against diseases such as cancer, aging and cardiovascular disease, while some have been reported to potentially offer protection from Alzheimer’s. Nonetheless, there is some evidence that the antioxidant compounds might not work *in vivo*. Some previous research findings based on observational epidemiology studies are contradicted by recently randomized trials [6]. For example, it is known that vitamin E has antioxidant activity; however randomized trials showed no benefit or even suggested increased harm [7]. Several and other contradictions persist [8,9,10]. However, the vast literature tells us that the plant-derived compounds, such as polyphenols besides being potent antioxidants, could also have the capability of modifying enzyme activity, interacting with molecules and altering the expression of important genes critical for human health as well as having a strong effect on oxidative stress reduction [11,12]. Also, it seems that while the human body has a diversity of antioxidant systems, there appear to be additional radicals that can be scavenged by the use of antioxidant products. Therefore significant studies are still needed in this subject, particularly those involving *in vivo* case studies area.

In addition, despite the good knowledge about potential effect of polyphenols and other compounds such as ascorbic acid content on antioxidant activity, the role of isothiocyanates and their precursors, (glucosinolates), in *Brassicaceae* plants is still scarce. It has been shown that the biochemical composition, either in profile and concentration, of *Brassica* products can vary widely, depending on the cultivar, fertilization and environment [13]. For instance, it is known that plants of the same cultivar, produced under similar agricultural conditions, could have significant variations in their bioactive components [14], with direct effects on human health, and yet these effects may also vary with the amount ingested and absorbed, which is somewhat dependent on the genetic characteristics of individuals [15].

With this study we aimed to take an integrated approach to study the antioxidant activity of the major bioactive components, e.g., phenolics, flavonoids, ascorbic acid and glucosinolates, present in the most consumed *Brassica* varieties, and to determine how this activity is influenced by the growth climate conditions. Putting together this information we further want to evaluate the antioxidant activity, a novelty of these kinds of studies, and we expect to find what the best cultivars, regarding such activity, are and how they are influenced by the climate conditions. Therefore, we intend, in an innovative way, add a better knowledge about each variety and what are the effects of different climate on nutritional value of *Brassica* vegetables, providing a clue for what is the best timing of harvest in terms of bioactivity.

## 2. Results and Discussion

### 2.1. Brassica bioactive compounds

Our results showed a variation of bioactive compounds (glucosinolates, total phenolics, total flavonoids and L-ascorbic acid), both in profile and content with genotype and climate season (Table 1 and Table 2). The GLS content in each *Brassica* sample showed significant differences (*P* < 0.05) between the two climate seasons Spring-Summer (SS) and Summer-Winter (SW) (Table 1); similar findings were noted for total flavonoids (*P* < 0.05) in all the *Brassica*, except for savoy cabbage, for L-ascorbic acid (*P* < 0.05) for all *Brassica* with the exception of turnip leaves, whilst the total phenolics content in the *Brassica* were generally less affected by climate seasons (Table 2).

It is clear the genotype × environment interaction in the levels of GLS, as described in other studies [7,16,17]. Ciska *et al.* [13] reported a year × cultivar interaction for total GLS in cruciferous vegetables, which they attributed to temperature and rainfall differences and the same observation was made in our study (Table 3). The average levels of individual GLS that were higher in the SW than in the SS seasons, along the two consecutive years were: glucobrassicin in broccoli inflorescences and in white cabbage; progoitrin and 4-methoxy-glucobrassicin in white cabbage; glucobrassicin in Portuguese tronchuda cabbage and finally glucobrassicanapin, gluconasturtiin and 4-hydroxyglucobrassicin in turnip roots. Whereas in the SS seasons they were: glucoiberin and neoglucobrassicin in Portuguese kale; glucoiberin in savoy cabbage and glucobrassicin in white cabbage. Our results thus indicate that GLS concentrations are seasonally affected in each botanical group. 

These results probably occurred due to genetic differences in GLS metabolism or storage, as reported by recent studies [18,19]. Besides the genetic factors, average temperatures and day length during the periods prior to harvest could be determinant on total and individual GLS concentrations, indicating that the effects of climatic conditions on GLS concentrations are especially critical. In general; our data show that total and main individual GLS were higher in SW season. In general, it seems that aliphatic and indole GLS are beneficed by climate conditions of SW. However, contrary to the expected, we found an opposite trend between years for total GLS in broccoli inflorescences and Savoy cabbage. The total GLS level was higher in SW season in 2005–2006, but higher in SS season in 2006–2007. This could be explained by the variation in some individual indole GLS, particularly 4-methoxyglucobrassicin and neoglucosbrassicin in broccoli and glucobrassicin in Savoy cabbage. 

**Table 1:** Mean content of GLS (µmoles·100 g^–1^ d.w.) in different *Brassica* samples produced in two consecutive years and two different climate seasons [Spring-Summer (SS) and Summer-Winter (SW)].^1,2,3,4^

Botanical group	Year	Climate season	Aliphatic						Aromatic	Indole				
			GIB	PROG	SIN	GNAP	GBRASSNAP	GRAF	GNAST	4-OH	GBRASS	4-METHOX	NGB	Total GLS
**Broccoli**	2005-2006	SS	120 ± 13	ND	ND	ND	ND	958 ± 68	ND	ND	175 ± 19	30 ± 3	579 ±132	1862 ± 182
		SW	ND	ND	ND	ND	ND	2152 ± 157^*^	ND	ND	529 ± 28^*^	173 ± 19^*^	273 ± 20^*^	3128 ± 124^*^
	2006-2007	SS	132 ± 4^*^	ND	ND	ND	ND	1154 ± 37	ND	64 ± 2 ^*^	350 ± 22	547 ± 17^*^	1694 ± 78^*^	3942 ± 159^*^
		SW	110 ± 5	ND	ND	ND	ND	1222 ± 65	ND	56 ± 1	589 ± 65^*^	434 ± 6	274 ± 34	2686 ± 50
**Portuguese kale**	2005-2006	SS	679 ± 88^*^	ND	368 ± 24	ND	ND	ND	ND	ND	272 ± 44	ND	47 ± 4 ^*^	1366 ± 103^*^
		SW	101 ± 7	17 ± 3	284 ± 20	ND	ND	25 ± 2	ND	ND	187 ± 10	34 ± 2	30 ± 1	676 ± 13
	2006-2007	SS	732 ± 10^*^	17 ± 5	328 ± 34^*^	ND	ND	43 ± 5	ND	12 ± 1	608 ± 73^*^	71 ± 12	45 ± 10^*^	1855 ± 112^*^
		SW	68 ± 22	ND	21 ± 11	ND	ND	60 ± 7	ND	34 ± 7 ^*^	107 ± 15	69 ± 10	8 ± 4	369 ± 53
**Savoy cabbage**	2005-2006	SS	718 ± 76^*^	59 ± 7	236 ± 18	ND	ND	ND	ND	ND	210 ± 28	ND	ND	1224 ± 85
		SW	355 ± 24	75 ± 4	329 ± 12^*^	ND	ND	222 ± 19	ND	ND	320 ± 2^*^	203 ± 11	11 ± 0^*^	1515 ± 35^*^
	2006-2007	SS	932 ± 58^*^	87 ± 6	149 ± 10	21 ± 1	ND	396 ± 41	ND	73 ± 7^*^	1144 ± 37^*^	611 ± 31	33 ± 5	3447 ± 51
		SW	865 ± 105	96 ± 22	290 ± 72	33 ± 5	ND	314 ± 54	ND	21 ± 6	832 ± 77	559 ± 24	18 ± 3	3030 ± 194
**White cabbage**	2005-2006	SS	106 ± 18	17 ± 2	106 ± 23	ND	ND	ND	ND	ND	20 ± 2	5 ± 0	2 ± 1	256 ± 46
		SW	174 ± 33	172 ± 14^*^	709 ± 38^*^	ND	ND	50 ± 8	ND	ND	219 ± 24^*^	119 ± 15^*^	16 ± 1^*^	1460 ± 18^*^
	2006-2007	SS	217 ± 15	33 ± 1	199 ± 23^*^	16 ± 2	ND	32 ± 3	ND	6 ± 2	137 ± 10	163 ± 28	26 ± 9	828 ± 57
		SW	452 ± 17^*^	236 ± 9^*^	63 ± 4	94 ± 20^*^	ND	958 ± 11^*^	ND	67 ± 6 ^*^	428 ± 25^*^	417 ± 19^*^	18 ± 3	2731 ± 33^*^
**Portuguese tronchuda cabbage**	2005-2006	SS	235 ± 19	ND	89 ± 12	ND	ND	ND	ND	ND	159 ± 27	6 ± 1	54 ± 6	543 ± 25
		SW	163 ± 32	23 ± 12	305 ± 50^*^	ND	ND	49 ± 19	ND	ND	252 ± 15^*^	ND	44 ± 2	836 ± 80^*^
	2006-2007	SS	571 ± 57	28 ± 5	232 ± 62	ND	ND	68 ± 13	ND	23 ± 2	537 ± 81	124 ± 6	135 ± 5	1718 ± 84
		SW	503 ± 76	36 ± 19	75 ± 41	ND	ND	289 ± 63^*^	ND	44 ± 3 ^*^	799 ± 34^*^	397 ± 36^*^	137 ± 12	2280 ± 52^*^
**Turnip leaves**	2005-2006	SS	ND	ND	ND	921 ± 41	876 ± 30	ND	71 ± 11	ND	16 ± 4	ND	54 ± 9	1937 ± 17
		SW	ND	ND	ND	848 ± 69	1067 ± 86	ND	71 ± 5	ND	62 ± 10^*^	96 ± 3	127 ± 4^*^	2270 ± 164
	2006-2007	SS	ND	ND	ND	1190 ± 40	1005 ± 100	ND	120 ± 11	9 ± 1	30 ± 2	ND	252 ± 5^*^	2606 ± 129
		SW	ND	ND	ND	2075 ± 568	1344 ± 101	ND	395 ± 58^*^	36 ± 16^*^	55 ± 11	ND	85 ± 24	3990 ± 403^*^
**Turnip roots**	2005-2006	SS	ND	ND	ND	538 ± 78	323 ± 57	ND	399 ± 69	ND	17 ± 3	ND	37 ± 8	1314 ± 212
		SW	ND	ND	ND	507 ± 68	583 ± 64^*^	ND	673 ± 104^*^	55 ± 11	34 ± 1^*^	ND	133 ± 6^*^	1984 ± 131
	2006-2007	SS	ND	ND	ND	394 ± 22	235 ± 28	ND	306 ± 14	57 ± 9	99 ± 11	134 ± 40	221 ± 13	1446 ± 109^*^
		SW	ND	ND	ND	872 ± 46^*^	572 ± 20^*^	ND	496 ± 20^*^	137 ± 2^*^	96 ± 10	1233 ± 74 ^*^	206 ± 20	3613 ± 103

^1^ Data are expressed as mean of three replicates ± error mean. ^2^ GIB-Glucoiberin; PROG-Progoitrin; SIN-Sinigrin; GNAP-Gluconapin; GBRASSNAP-Glucobrassicanapin; GRAF-Glucoraphanin; GNAST-Gluconaturtiin; 4-OH-4-hydroxyglucobrassicin; GBRASS-Glucobrassicin; 4-METHOX-4-Methoxyglucobrassicin; NGB-Neoglucobrassicin. ^3^ ND-Non detectable. ^4^ Mean of each compound for different climate season within same year were compared by Mann Whitney test, * P < 0.05.

**Table 2:** Mean content of total phenolics, total flavonoids and L-ascorbic acid in different *Brassica* samples produced in two consecutive years and two different climate seasons [Spring-Summer (SS) and Summer-Winter (SW)].^1,2^

Botanical group	Year	Climate season	Total phenolics (mg.g^–1^ GAE)	Total Flavonoids (mg.g^–1^ CAE)	L-ascorbic acid (mg.g^–1^ LACE)
**Broccoli**	2005-2006	SS	15.3 ± 0.8	7.7 ± 0.02 ^*^	98.2 ± 0.20 ^*^
		SW	13.5 ± 0.3	4.9 ± 0.10	97.3 ± 0.07
	2006-2007	SS	21.1 ± 0.3	12.1 ± 0.02 ^*^	96.3 ± 0.20 ^*^
		SW	24.3 ± 1.1 ^*^	3.3 ± 0.01	95.5 ± 0.05
**Portuguese kale**	2005-2006	SS	23.0 ± 1.0 ^*^	9.3 ± 0.02 ^*^	139.6 ± 0.06 ^*^
		SW	18.8 ± 0.7	7.4 ± 0.62	139.3 ± 0.04
	2006-2007	SS	27.4 ± 3.6	12.0 ± 0.02 ^*^	139.0 ± 0.06 ^*^
		SW	20.7 ± 0.5	8.1 ± 0.02	138.8 ± 0.01
**Savoy cabbage**	2005-2006	SS	9.9 ± 0.5	2.7 ± 0.28	107.5 ± 0.17 ^*^
		SW	12.9 ± 2.3	3.1 ± 0.20	106.7 ± 0.04
	2006-2007	SS	13.6 ± 0.1	4.1 ± 0.01	105.9 ± 0.17 ^*^
		SW	16.1 ± 1.4	4.1 ± 0.02	105.3 ± 0.06
**White cabbage**	2005-2006	SS	11.1 ± 1.5	3.0 ± 0.02	70.2 ± 0.09
		SW	13.0 ± 1.1	6.5 ± 0.01 ^*^	71.6 ± 0.30 ^*^
	2006-2007	SS	8.7 ± 0.1	2.5 ± 0.02	67.7 ± 0.08
		SW	14.8 ± 0.7 ^*^	3.2 ± 0.00 ^*^	68.8 ± 0.30 ^*^
**Portuguese tronchuda cabbage**	2005-2006	SS	15.7 ± 1.1	2.1 ± 0.01	138.9 ± 0.03
		SW	19.0 ± 0.9	6.9 ± 0.02 ^*^	139.1 ± 0.06 ^*^
	2006-2007	SS	24.8 ± 0.8 ^*^	10.5 ± 0.02 ^*^	138.6 ± 0.06 ^*^
		SW	18.6 ± 0.5	5.0 ± 0.02	138.4 ± 0.03
**Turnip leaves**	2005-2006	SS	15.3 ± 1.0	3.5 ± 0.01	132.3 ± 0.04
		SW	19.5 ± 0.7 ^*^	5.6 ± 0.11 ^*^	132.7 ± 0.08
	2006-2007	SS	17.1 ± 0.5	5.5 ± 0.02	131.6 ± 0.02
		SW	15.8 ± 8.6	8.7 ± 0.01 ^*^	131.9 ± 0.08
**Turnip roots**	2005-2006	SS	8.8 ± 1.1	0.7 ± 0.06	22.9 ± 0.13
		SW	11.8 ± 0.3	7.6 ± 0.06 ^*^	25.0 ± 0.46 ^*^
	2006-2007	SS	7.3 ± 0.2	1.6 ± 0.01	18.9 ± 0.13
		SW	10.5 ± 0.3 ^*^	2.0 ± 0.01 ^*^	20.6 ± 0.46 ^*^

^1^ Data are expressed as mean of three replicates ± error mean; ^2^ Mean of each compound for different climate season within same year were compared by Mann Whitney test, * P < 0.05.

**Table 3:** Evolution of minimum average temperature (T_min_), maximum average temperature (T_max_), mean average temperature (T_mean_) and average rainfall (mm) in Vila Real region (Northern Portugal), during the entire cycle of *Brassica* production. Comparison with the average levels in the last 30 years.

Month	1961–1990				2005–2006				2006–2007			
	T min (°C)	T max (°C)	T mean (°C)	R (mm)	T min (°C)	T max (°C)	T mean (°C)	R (mm)	T min (°C)	T max (°C)	T mean (°C)	R (mm)
**March**	4.8	14.4	9.6	3.1	5.3	15.7	10.5	1.8	6.2	14.1	10.2	5.3
**April **	6.4	16.5	11.4	2.9	7.3	17.1	12.2	1.7	8.2	19.5	13.8	2.0
**May**	8.9	20.1	14.5	2.3	9.7	21.8	15.7	1.4	10.3	23.7	17.0	0.2
**June**	12.4	25.0	18.7	1.8	15.0	28.4	21.7	0.1	13.8	27.2	20.5	2.5
**July**	14.3	28.8	21.6	0.5	15.0	28.9	22.0	0.3	15.8	30.3	23.1	0.9
**August**	13.8	28.7	21.3	0.5	16.3	31.4	23.8	0.1	15.4	29.7	22.6	1.0
**September**	12.6	25.7	19.2	1.6	12.8	25.9	19.3	0.8	13.7	26.1	19.9	3.1
**October**	9.4	19.5	14.4	3.5	10.9	19.8	15.4	4.6	11.8	19.7	15.7	6.1
**November**	5.4	13.5	9.5	4.2	5.0	12.5	8.8	2.2	8.6	15.0	11.8	7.2
**December**	3.3	10.0	6.7	5.2	2.8	10.3	6.6	3.8	3.0	9.9	6.5	3.9
**January**	2.6	9.7	6.2	5.2	1.6	8.6	5.1	1.0	3.3	10.5	6.9	0.6

The variations of temperature, water stress or radiation prior and/or during the harvest, could justify the differences found. Moreover, it seems that the GLS concentrations will be dependent of the cultivar adaptation to climate factors variation and therefore, not all cultivars are affected in same way.

For the total phenolics, total flavonoids and L-ascorbic acid, due to our geographic and climate conditions, it seems that for broccoli inflorescences (cv. Marathon) and Portuguese kale the SS conditions caused an increase in total flavonoids and L-ascorbic acid, probably caused by high temperatures and radiation (not shown) and lower precipitation (Table 3) and therefore higher water stress, particularly in the days near harvest time, which was noted also in previous reports for other species [20,21,22,23]. An opposite trend was observed for flavonoids and L-ascorbic acid in white cabbage, where the levels of these bioactives were higher in SW than SW, in the two consecutive years. Based on these results total flavonoids and L-ascorbic acid contents in *Brassica* plants are affected by climate, but their average is dependent of cultivar.

For turnip, climate season affected the average content of total flavonoids in leaves and roots, and L-ascorbic acid only in the roots (Table 2). The levels of these two bioactive compounds, in this group, were high in SW seasons (Table 2), which seems to confirm the same idea stated for the GLS levels, *i.e*., plants with short production cycles, such as turnip, accumulate higher levels of bioactive compounds due to synthesis stimulation induced by moderate temperatures and considerable radiation at the beginning of production cycle (August-September-October), compared to the equivalent SS season period (March-April-May; Table 1, Table 3). Less significant variation was noted in total phenolics content; nevertheless, the levels in roots were consistently higher in SW face to SS seasons, probably due to the same reason pointed for total flavonoids and L-ascorbic acid content. Instead, the average levels of total phenolics in the leaves were significant higher in SW seasons, only in the first year (Table 2).

### 2.2. Effect of climate and bioactive components on antioxidant activity

Previous studies have shown that very few glucosinolates, or their hydrolysis products, have direct antioxidant effects [24,25]. There are many studies showing indirect antioxidant activity due to the induction of genes associated with various redox mechanisms in cells [26,27]. The only glucosinolates, and corresponding hydrolysis products, that have significant direct antioxidant activity are glucoerucin (4-methylthiobutyl GLS) and glucoraphasatin (4-methylthio-3-butenyl GLS) [28]. Glucoerucin is found in many *Brassicacea*/*Brassica* species (e.g., high levels in seeds of broccoli and rockets) and glucoraphasatin is present in fewer non-*Brassica* Brassicaceae species e.g. *Bunias*, *Raphanus* and *Matthiola* [29,30,31]. 

**Table 4:** % Inhibition of DPPH radicals at 5 mg·mL^–1^ and IC_50_ (mg·mL^–1^) values determined from the dose-curve response for each plant extract in two consecutive years and two different climate seasons [Spring-Summer (SS) and Summer-Winter (SW)].^1,2^

Plant extract	Year	Climate season	% Inhibition DPPH radicals	IC_50_ (mg·mL^-1^)
**Broccoli**	2005-2006	SS	89.3^*^	1.47
		SW	84.9	2.56
	2006-2007	SS	70.2	1.6
		SW	83.0^*^	2.23
**Portuguese kale**	2005-2006	SS	91.6^*^	1.10
		SW	84.2	1.10
	2006-2007	SS	83.8^*^	1.65
		SW	62.7	2.14
**Savoy cabbage**	2005-2006	SS	54.0	4.61
		SW	68.9^*^	3.49
	2006-2007	SS	47.9	5.48
		SW	59.8^*^	4.4
**White cabbage**	2005-2006	SS	27.2	8.14
		SW	37.9^*^	6.95
	2006-2007	SS	33.6	>4.27
		SW	68.1^*^	3.74
**Portuguese tronchuda cabbage**	2005-2006	SS	88.8	1.48
		SW	89.8^*^	2.12
	2006-2007	SS	79.1^*^	1.34
		SW	71.1	2.93
**Turnip leaves**	2005-2006	SS	77.2	2.22
		SW	91.2^*^	1.32
	2006-2007	SS	46.4	9.19
		SW	74.7^*^	2.12
**Turnip roots**	2005-2006	SS	14.1	9.18
		SW	28.8^*^	7.98
	2006-2007	SS	26.5	>4.82
		SW	49.3^*^	5.68
**Control**		Trolox	89.9	0.24

^1^ Data are expressed as mean of three replicates; ^2^ Mean of each compound for different climate season within same year were compared by Mann Whitney test, * P < 0.05.

Neither of the two GLS with direct antioxidant activity was detected in the species used in the current study; therefore there are no direct correlations between GLS (total or individual) and direct antioxidant activity. The antioxidant activity (AA) is expressed as % inhibition of DPPH radicals at 5 mg·mL^–1^ of plant aqueous extracts and the IC_50_ values (Table 4) were calculated from the dose-dependent curves (not shown). Among the different *Brassica* extracts, the broccoli inflorescences and Portuguese tronchuda cabbage exhibited the highest AA, with 82% of inhibition of DPPH radicals, followed by Portuguese Kale with 81% of inhibition of DPPH radicals. The turnip leaves, Savoy-cabbage, white cabbage and turnip roots exhibited lower AA with approximately 72%, 58%, 42% and 30% of inhibition of DPPH radicals at 5 mg·mL^–1^, respectively (Table 4).

When we compare these results with the achieved for the commercial positive control Trolox (Table 4) we noted that only two values, from Portuguese kale and turnip leaves, showed a % inhibition of DPPH radicals higher than the control. Nevertheless, in the global results, a total of nine values showed a % DPPH radical’s inhibitions ≥92% relative to the control. Independently of year and climate season, the turnips showed the lowest values of % inhibition of DPPH radicals. These results are corroborated in some way by the IC_50_ values. The lowest average IC_50_ value (mg·mL^–1^) and therefore the higher average AA was founded for the Portuguese kale (1.5 mg·mL^–1^, Table 4), whereas the turnip roots showed the highest IC_50_ average value (8 mg·mL^–1^, Table 4). It seems that AA activity could be related with genotype due to the differences occurred between different botanical groups. Experiments with different kind of vegetables [32] showed that, among different plants, broccoli inflorescences presented the highest AA (73–79%) when compared with tomato (15–60%), spinach (40–60%) and potato (13–38%). The current study shows that in Portuguese kale there was an increase of AA in the SS season, in the two consecutive years. The same results was observed, but for SW season, in Savoy cabbage, white cabbage and turnip (leaves and roots). Despite the same trend noted for the turnip group, the potential AA in the leaves is higher than in the roots.

Several previous studies have evaluated the relationship between the AA and several antioxidant compounds, such as phenolics, flavonoids and vitamin C. [1,32,33] In order to clarify which group of compounds mainly contributes to the AA, we evaluated the relationship between the content of total phenolics, total flavonoids, L-ascorbic acid and the AA (expressed by % inhibition of DPPH radicals), showed by the different *Brassica* extracts. To study that, principal component analysis (PCA), Pearson’s correlation coefficient and Spearman Rank order correlations were performed and analysed.

**Figure 1:**
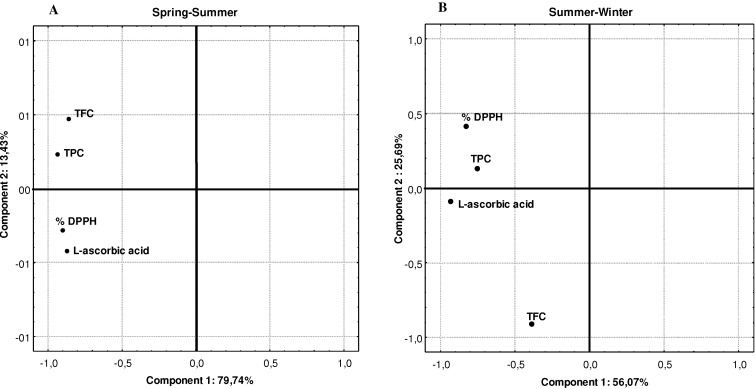
PCA results for AA versus % DPPH: A- across all *Brassica* samples harvested in Spring-Summer season; B- across all *Brassica* samples harvested in Summer-Winter season.

**Figure 2:**
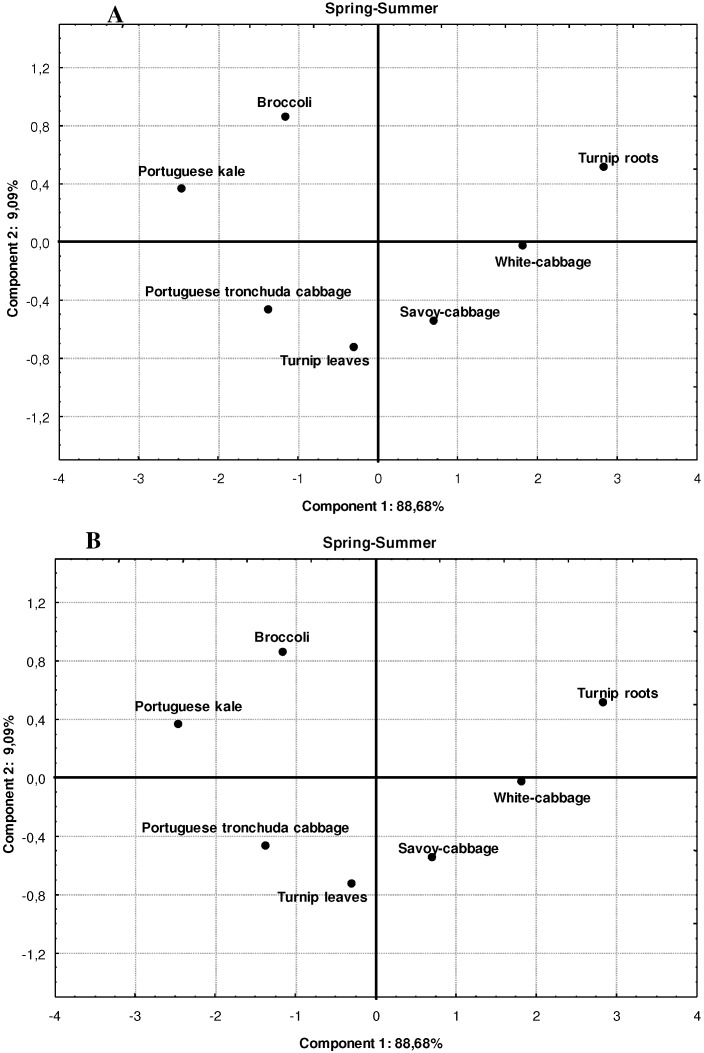
Principal component analysis (PCA) results, aggregating all *Brassica* samples.

Based on previous studies it is expected that there would be a direct relationship between relationship between the AA, expressed as % inhibition of DPPH radicals, and the average content of the different antioxidant compounds. The results are presented in Table 5 and Figure 1 and Figure 2.

**Table 5:** Significant correlations found between % Inhibition DPPH radicals and selected bioactive components.^1^

Compounds	Pearson’s correlation coefficients
Total phenolics vs DPPH	0.6400 **
Total flavonoids vs DPPH	0.4580 **
L-ascorbic acid vs DPPH	0.7620 **

^1^ Number of samples (N) was 84; ** P<0.01.

The results achieved with Pearson´s correlation shown that the % inhibition of DPPH radicals were more associated with L-ascorbic acid content than TPC and TFC (Table 5), which means that *Brassica* with higher content of L-ascorbic acid probably presents higher AA. It seems that AA is more dependent on effects of plant part/species rather than climate variations. Nonetheless, the climatic factors are very important because they can interfere in the chemical biosynthesis and therefore on the final concentration of bioactive compounds [23], thus interfering in the antioxidant properties of vegetables, as it seems to be the current situation. In the current work, it was noted that in climate seasons in which L-ascorbic acid and, to a minor extent, the two other bioactive compounds, were increased, the AA increased. Also, the Sperman rank order correlation was 0.7207(***) for L-ascorbic acid and % DPPH, 0.6529(**) for % DPPH and TPC; and 0.4830(**) for % DPPH and TFC, which confirms one more time the preponderance of L-ascorbic acid on AA. However, when we perform the PCA, individually, for SS and SW climate seasons, we noted some differences. In SS the AA followed the same trend descript above (Figure 1B), but in SW (Figure 1C) the major influence on AA was from TPC plus L-ascorbic acid. The variation of these two compounds explained about 81.7% of total variance in this season, whilst in SS the increment of AA was explained with 93.2% of total variance with increment of L-ascorbic acid. In both cases less influence were noted for TFC. These results seem to confirm the idea that L-ascorbic acid in fact is very important as antioxidant compound.

Another interesting result with PCA, is when we apply the method across all *Brassica* samples, in order to aggregate them in different climate periods (Figure 2A,B), we verify that in SS season the broccoli and Portuguese kale can be aggregated in one group, Portuguese tronchuda cabbage and turnip leaves in another (Figura 2A), whilst in SW the Portuguese trocnhuda, turnip leaves and Portuguese kale can be combined in another. This tendency could reflect the higher content of TFC and L-ascorbic acid in SS for broccoli and Portuguese kale, whilst for SW season the similar content of TPC, TFC and L-ascorbic acid between those three *Brassica* can explain their proximity. Therefore based on these results it seems that on SS season the broccoli inflorescences and Portuguese kale can be an important source of natural antioxidants, whilst in SW that source could be the turnip leaves, Portuguese tronchuda cabbage and also, Portuguese kale. The savoy-cabbage, white-cabbage and turnip roots are less important as a source of antioxidant compounds, nonetheless their importance must not be depreciated due to some considerable amounts of bioactive compounds particularly in SW. Based on these results it is clear that not all of the bioactive components are critical for the AA, nonetheless it seems that the AA is often associated with a combined/synergistic, which is partially in agreement with several authors, [33,34,35] who found strong correlations between the AA and the combined effect of total flavonoids and vitamin C.

Finally, another important aspect within the scope of these results is related to the validation of PCA analysis method as an instrument to study the different contribution of the different bioactive components to AA. Based on the current results, it seems that PCA analysis is a valid method, due to the strong correlations found between the variations of AA and the variations of average content of the different biological components. This analysis allowed establishing logical patterns between variations of AA and the variation of the different bioactive components. 

## 3. Experimental

### 3.1. Plant material

**Figure 3:**
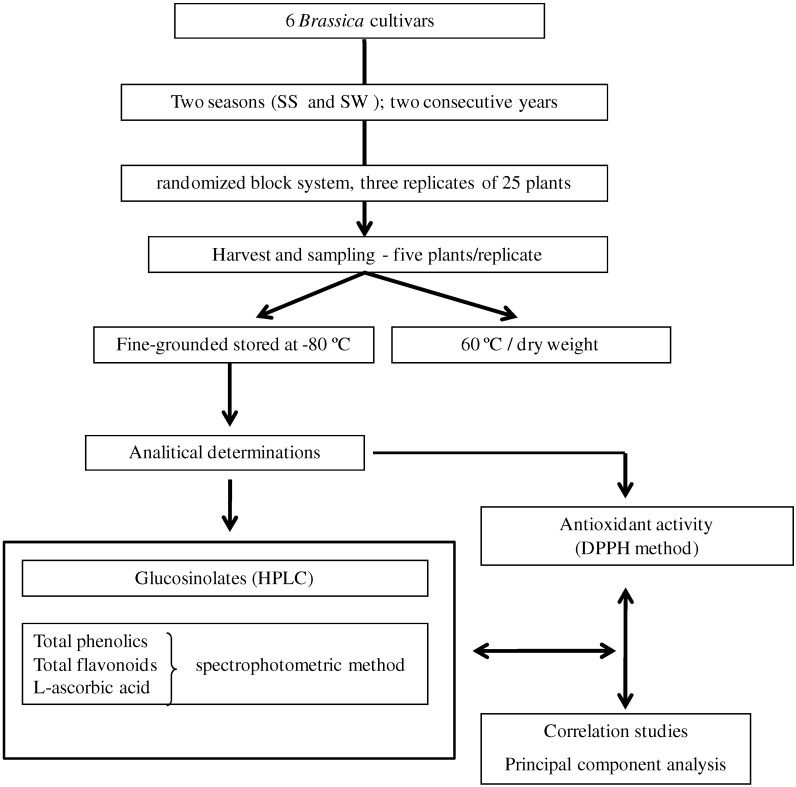
Main steps of experimental design applied in this work.

Experiments were conducted in two growing seasons Spring-Summer (SS) and Summer-Winter (SW) for two consecutive years in Vila Real region, Northern Portugal (460 m altitude, 41°17´N and 7°44´W). The analytical procedure adopted is briefly represented below in diagram as Figure 3. 

For this study six widely consumed *Brassica* vegetables from three botanical groups were selected: one represented by broccoli inflorescences (*Brassica oleracea* L. var. italic Plenck cv. Marathon); a second group made up of leafy-type ones, Portuguese kale (*Brassica oleracea* L. var. *acephala* D.C), white cabbage (*Brassica oleracea* L. var. *capitata* alba Alef.), savoy cabbage (*Brassica oleracea* L. var. *sabauda* L.) and Portuguese tronchuda cabbage (*Brassica oleracea* L. var. *costata* D.C.); and a root-type (white tip), a turnip (*Brassica rapa* L.*rapa* cv. Nabo da horta). Plants were started in pots, in a greenhouse, after sowing in a mixture of sterilized peat and sand (3:1, ^v^/_v_). They were transplanted to the field at 3-4 true leaf stage and watered and irrigated when necessary. The soil was previously analyzed in Soil department of University of Trás-os-Montes e Alto Douro for nutrients balance and no major requirements were indicated, the nutritional level were adequate to *Brassica* cultivation, so no fertilizations were made during the experiment. Also, no pesticides were applied. The experiment was set in a randomized block system, with three replicates of 25 plants each. The sowing dates were 24 March 2005 and 18 April 2006 for SS seasons and 19 August 2005 and 14 August 2006 for SW seasons. Plant material was harvested at commercial maturity stage, and counted as the number of days after transplantation; in SS between 67–68 days for broccoli inflorescences, 52–54 days for Portuguese kale, 66–67 days for white, Savoy and Portuguese tronchuda cabbage, 42–45 and 52–53 days for turnip leaves and roots, respectively; in SW the harvest occurred, between 95–98 days for broccoli inflorescences, 68–80 days for Portuguese kale, 126–130 days for savoy cabbage, and 87–95 days for white cabbage and Portuguese tronchuda cabbage, 39–49 and 66–68 days for turnip leaves and roots, respectively. During the growing season climate data were recorded (as shown in Table 3). At commercial maturity stage were harvested five plants from each replicate, always at the same time of the day to avoid diurnal influences. About half of the plant material of each replicate was immediately frozen in liquid nitrogen, fine-ground in a mortar and stored at –80 °C until analyses; the remaining half was dried in a forced-air oven at 60 °C (Memmert UL 80, Germany), for dry weight (d.w.) evaluation.

### 3.2. Determination of bioactive compounds

#### 3.2.1. Glucosinolates

Glucosinolates (GLS) extraction was done according to Rosa [36] and Pereira *et al.* [37]. Samples were freeze-dried (Dura-Dr^TM^ µP-FTS Systems) and fine-ground; then 0.2 mg d.w. were extracted with boiling methanol [90% (v/v), 3 mL] for 2 minutes. A solution of benzyl GLS (glucotropaeolin) 50% (m/v) was used as internal standard. After re-extraction with boiling 70% (v/v) methanol, the supernatant were combined to a final volume of 10 mL. An aliquot (2.5 mL) was evaporated to dryness and resuspended in water (2.5 mL) and 2 mL was applied to a small column of DEAE Sephadex A25 as described previously [38]. Desulpho-GLS were obtained using commercial aryl sulphatase (EC 3.1.6.1) Type H1 from *Helix pomatia* (Sigma Chemical Co., St. Louis, MO, USA) at 14.6 Units g^–1^. The desulpho-GLS were eluted with water and analyzed using high performance liquid chromatography (HPLC) [39]. The procedure adopted corresponds to the ISO 9167-1 method (EEC Regulation No. 9167-1, 1992). GLS peak identification and quantitative estimations were made using pure standard GLS as internal standard (benzyl GLS), and response factors. GLS concentrations were expressed in μmol g^–1^ dry weight (d.w.). All reagents were of analytical or HPLC grade. 

#### 3.2.2. Total phenolics (TP) and total flavonoid contents (TF)

For TP determination the method involving Folin-Ciocalteu reagent and gallic acid as standard was used [21,40]. In brief, dry plant material (5 g) was homogenized (Mod.F60, Falc Intruments, Italy) in bi-distilled water (250 mL) kept at 100 °C until ebullition and then filtered. The plant extract was then resuspended in bi-distilled water and supernatant (1 mL) was mixed with Folin - Ciocalteu’s solution (Panreac, Spain, 1:10 ^v^/_v,_ 1 mL) and Na_2_CO_3_ solution (Sigma-Aldrich, 7% ^W^/_V,_ 10 mL). After 90 minutes in the dark at room temperature, absorbance was measured at 765 nm (U-2000, serial 121-0120. Hitachi Ltd., Japan). Standard gallic solutions (0–10 mg mL^–1^) were also assayed and calibration curve was obtained from the equation: y = 5.703x + 0.089; R^2^ = 0.996. A blank assay, using bi-distilled water, was also prepared. The phenolic contents were expressed as mg g^–1^ dry weight (d.w.) GAE (Gallic acid equivalent). Samples were analyzed in triplicate.

Te TF determinations were made using colorimetric assays [41,42]. A mixture of diluted extracts (1 mL), bi-distilled water (4 mL) and NaNO_2_ solution (Sigma-Aldrich, 5% ^W^/_V,_ 0.3 mL) was prepared After shaking and 5 minutes at room temperature, AlCl_3_ solution (Sigma-Aldrich, 10% ^W^/_V,_ 0.3 mL) was added and allowed to stand 6 minutes. After, NaOH (Sigma-Aldrich, 1 M, 2 mL) was added. Finally, volume was completed to 10 mL with bi-distilled water and thoroughly mixed. The absorbance of the pink mixture was measured at 510 nm. Standard catechin (Sigma-Aldrich) solutions (0–10 mg mL^–1^) were also essayed and calibration curve was obtained from the equation: y = 0.029x + 0.188; R^2^ = 0.993. A blank essay, using bi-distilled water, was also prepared. Total flavonoids were expressed as mg g^–1^ d.w. CE (catechin equivalent). Total flavonoids determinations were made in triplicate.

#### 3.2.3. L-Ascorbic acid

The content of ascorbic acid was determined by the colorimetric assays [35] with some modifications [43,44]. Briefly, for a sample of 20 mg a metaphosphoric acid solution [(HPO_3_)_n_, Fluka, 5% ^V^/_V_, 10 mL) was added and then homogenized and allowed to stand at room temperature during 45 minutes with permanently agitation (Mod. F60, Falc Instruments, Italy). Extracts were filtered and the ascorbic acid assay was performed on supernatant (1 mL) with 2.6 dicloroindophenol solution [DIP, C_12_H_6_C_l2_NNaO_2_•xH_2_O, Fluka, 25% of DIP and 21% NaNO_2_^W^/_V,_ 9 mL). After 15 seconds absorbance was measured at 515 nm using a spectrophotometer. Standard L-ascorbic acid (C_6_H_8_O_6_, Aldrich) was prepared and a calibration curve was obtained from the following equation: y= –0.025x + 3.8861; R^2^ = 0.9926. The average content was expressed as mg g^–1^ d.w. LACE (L-ascorbic acid equivalent). All determinations were made in triplicate.

### 3.3. Determination of antioxidant activity

The antioxidant activity was measured by the 2'-diphenyl-1-picryl hydrazyl (DPPH radical scavenging activity) method. This method involves a determination of inhibition activity of antioxidant against stable DPPH (2'-diphenyl-1-picrylhydrazyl) radical which is determined spectrophotometrically [45]. Briefly, stock solutions of crude extracts were prepared as 0.25, 0.5, 1.0, 1.25, 2.5, 5.0 and 10 mg mL^–1^ in water. Antioxidant activity was measured using 300 μL of each previous concentration and 2.7 mL of 2'-diphenyl-1-picryl hydrazyl (DPPH) solution, 95% in ethanol (^W^/_V_), followed by agitation and reaction in dark at room temperature during 60 minutes. Absorbance values were measured at 517 nm. A blank assay, using water, was also prepared. A positive control was made with standard solution Trolox (C_14_H_18_O_4_, Sigma-Aldrich) in same concentrations than crude extracts. Radical scavenging activity was calculated by the following formula:

Inhibition = [(Abs_blank_ – Abs_sample_ (t) / Abs_blank_] × 100

where Abs_blank_ is absortion of blank sample (t = 0 minutes); Abs_sample_ (t) is absortion of tested extract solution (t = 60 minutes). The IC_50_ (“*half maximal effective concentration*”) values were calculated based on a dose-response curve, where abscissa was concentration of tested plant extracts and ordinate the average inhibition percentage. The experiment was carried out in triplicate.

### 3.4. Statistical analysis

For statistical analyses was used the SPSS V.17 statistical package (SPSS, Inc. Chicago, Illinois, USA). The results are presented as mean of three replicates. ANOVA was used to compare differences in GSs concentrations in different brassicas between seasons and years and Mann Whitney test was used to compare means. Bilateral correlations were determined by Pearson correlations coefficient and Spearman´s rank coefficient was used to determine associations between the different parameters measured, across all species. It was also performed a principal component analysis (PCA) in order to identify patterns between antioxidant activity and different growing/climate season. The level of significance was set at 0.05.

## 4. Conclusions

*Brassica* aqueous extracts have shown an interesting antioxidant potential and therefore may constitute an important natural source of antioxidants. Portuguese kale was the *Brassica* spp. with the highest antioxidant activity in the SS season, whilst in SW the highest AA was exhibited by turnip leaves, mainly due to their content of L-ascorbic acid but also, even if in a less extent, of TPC and TFC. The antioxidant potential can be affected by climate conditions, due to their influence on the average content of bioactive components. Thus, the climate during the growth period of the plants is extremely important and different factors such as temperature, precipitation and radiation can influence the accumulation of several chemical components and consequently affecting the antioxidant properties of *Brassica* vegetables.
